# Targeting In-Stent-Stenosis with RGD- and CXCL1-Coated Mini-Stents in Mice

**DOI:** 10.1371/journal.pone.0155829

**Published:** 2016-05-18

**Authors:** Sakine Simsekyilmaz, Elisa A. Liehn, Stefan Weinandy, Fabian Schreiber, Remco T. A. Megens, Wendy Theelen, Ralf Smeets, Stefan Jockenhövel, Thomas Gries, Martin Möller, Doris Klee, Christian Weber, Alma Zernecke

**Affiliations:** 1 Institute for Molecular Cardiovascular Research, University Hospital Aachen, RWTH Aachen University, Aachen, Germany; 2 Institute of Technical and Macromolecular Chemistry, RWTH Aachen University, Aachen, Germany; 3 Biochemistry Institute, Justus-Liebig-University, Giessen, Germany; 4 Human Genetic Laboratory, University of Medicine and Pharmacy, Craiova, Romania; 5 IZKF Aachen, University Hospital Aachen, RWTH Aachen University, Aachen, Germany; 6 Department of Applied Medical Engineering, Helmholtz Institute for Biomedical Engineering, RWTH Aachen University, Aachen, Germany; 7 Institute for Textile Technology, RWTH Aachen University, Aachen, Germany; 8 Institute for Cardiovascular Prevention, Ludwig-Maximilians University Munich, Munich, Germany; 9 Department of Oral and Maxillofacial Surgery, Center of Clinical Neurosciences, University Medical Center Hamburg-Eppendorf, Hamburg, Germany; 10 Institute for Experimental Biomedicine, University Hospital Würzburg, Würzburg, Germany; William Harvey Research Institute, Barts and The London School of Medicine and Dentistry, Queen Mary University of London, UNITED KINGDOM

## Abstract

Atherosclerotic lesions that critically narrow the artery can necessitate an angioplasty and stent implantation. Long-term therapeutic effects, however, are limited by excessive arterial remodeling. We here employed a miniaturized nitinol-stent coated with star-shaped polyethylenglycole (star-PEG), and evaluated its bio-functionalization with RGD and CXCL1 for improving in-stent stenosis after implantation into carotid arteries of mice. Nitinol foils or stents (bare metal) were coated with star-PEG, and bio-functionalized with RGD, or RGD/CXCL1. Cell adhesion to star-PEG-coated nitinol foils was unaltered or reduced, whereas bio-functionalization with RGD but foremost RGD/CXCL1 increased adhesion of early angiogenic outgrowth cells (EOCs) and endothelial cells but not smooth muscle cells when compared with bare metal foils. Stimulation of cells with RGD/CXCL1 furthermore increased the proliferation of EOCs. *In vivo*, bio-functionalization with RGD/CXCL1 significantly reduced neointima formation and thrombus formation, and increased re-endothelialization in *apoE*^*-/-*^ carotid arteries compared with bare-metal nitinol stents, star-PEG-coated stents, and stents bio-functionalized with RGD only. Bio-functionalization of star-PEG-coated nitinol-stents with RGD/CXCL1 reduced in-stent neointima formation. By supporting the adhesion and proliferation of endothelial progenitor cells, RGD/CXCL1 coating of stents may help to accelerate endothelial repair after stent implantation, and thus may harbor the potential to limit the complication of in-stent restenosis in clinical approaches.

## Introduction

Cardiovascular disease is the most frequent cause of death in industrialized nations. Atherosclerosis as the underlying disease [1,2] can result in a narrowing of the artery, necessitating angioplasty and stent-implantation. Long-term effects of such therapy, however, are limited by arterial remodeling and restenosis, and the risk of life-threatening stent-thrombosis [3,4]. While meta-analyses have shown no differences in stent thrombosis comparing drug-eluting with bare-metal stents [5–7], there is also evidence of an increased risk of very late stent thrombosis with drug-eluting stents [6,8–10], possibly related to reduced vessel wall re-endothelialization [11–13].

Different factors contribute to endothelial repair and plaque formation. The chemokine CXCL1 enhances re-endothelialization and reduces neointima formation, and its receptor CXCR2 mediates homing of circulating endothelial progenitor cells to sites of arterial injury in mice [14–16]. Furthermore, stents coated with cRGD (Arg-Gly-Asp)-peptide, which preferentially bind αvβ3 and α5β-integrins, attract endothelial progenitor cells to stented areas, accelerating wound healing in swine [17].

Thus, RGD and CXCL1 may serve as candidates for the coating of stents to enhance re-endothelialization after implantation. We here employed a miniaturized stent implanted into the carotid artery of mice [18], and evaluated its bio-functionalization. Indeed, we could show that the bio-functionalization of star-PEG-coated nitinol-stents with RGD/CXCL1 supported the adhesion and proliferation of endothelial progenitor cells and thereby reduced in-stent neointima formation.

## Materials and Methods

### Cell culture

Isolation and cell culture of early angiogenic outgrowth cells (EOCs) were performed as described [14,19–22]. Briefly, peripheral blood mononuclear cells were separated by Biocoll density gradient centrifugation (Biochrom) from buffy coats derived from healthy human donors and plated on fibronectin-coated (10μg/ml) 6-well plates (1x10^7^ cells/well) in endothelial growth medium MV2 (PromoCell), changed at day 1 and 4, and harvested at day 7. Under these conditions, cultured cells developed a spindle-shaped appearance, formed typical cell clusters and bound endothelial-specific lectin, identifying these as early angiogenic outgrowth cells. Human umbilical vein endothelial cells (HUVECs, PromoCell, Cat. No. C-12200) were cultured in endothelial cell growth medium and used between passages 3 to 6. Human coronary artery smooth muscle cells (SMCs, PromoCell, Cat. No. C-12511) were cultured in smooth muscle cell growth medium 2 (PromoCell) and used between passages 3 to 6. Adherent cells were detached for experiments by applying accutase (PAA Laboratories) for 5 minutes at 37°C.

### Stent braiding- and nitinol-coating

Stents or nitinol foils were processed as previously described [18]. Stents were manually braided using 16 nitinol wires (50μm diameter) yielding an outer dimension of 500μm, and heated in a high-temperature oven for shape settings. Stents or nitinol foils were cleaned by sonication in acetone, water, and 2-propanol followed by drying in a nitrogen stream. Surface activation was achieved by treatment with UV/ozone before use for aminofunctionalization. Substrates were immersed in a solution of N-[3-(trimethoxysilyl)-propyl]ethylenediamine in dry toluene, and the desired amount of isocyanate end group terminated six-arm star-shaped copolymer of 80% ethylene oxide and 20% propylene oxide NCO-sP(EO-*stat*-PO) (Mn = 12,000g/mol; PD = 1.15) was dissolved in dry tetrahydrofurane under an inert gas atmosphere. Thereafter, the solution was filtered through 0.2 μm syringe filters and used for hydrogel coating (5 min) of stents or foils. Subsequently, biofunctionalization was achieved by overnight incubation in 300μl polymer solution containing 50μg/ml RGD or 50μg/ml RGD and 1μg/ml CXCL1 in PBS, followed by extensive washing in water.

### Adhesion assay under static conditions

For adhesion assays under static conditions, 1x10^5^ cells were seeded on nitinol foils coated with star-PEG with/without RGD and/or CXCL1. After an overnight incubation and 2 washing steps with 1x Dulbecco’s PBS (PAA), adherent cells were detected by staining with DAPI and visualized with a Leica DM-RXE fluorescence microscope. Adherent cells were quantified in 15 recorded high power fields per sample using Diskus software.

### Proliferation and viability assay

Proliferation assays were performed in untreated cells, or after stimulation with RGD or a combination of RGD together with CXCL1 using the BrdU Cell Proliferation Assay, HTS (Calbiochem), according to the manufacturers’ instructions using 1x10^5^ cells/well in 96-well plates. Viability was tested using the fluorescent CellTiter-Blue^®^ cell viability assay (Promega), according to the manufacturers’ instructions.

### Stent implantation and Immunohistochemistry

Stents were implanted into left carotid arteries of anesthetized (100 mg/kg ketamine hydrochloride and 10 mg/kg xylazine intraperitoneally) mice by inserting 500 μm silicon tubes containing the stent through an incision in the external carotid artery, and careful forward-feeding of the stent whilst retraction of the tube allowing for shape memory-based expansion [18]. In total, 38 mice underwent stent implantation but 6 mice died during the procedure and therefore were not considered for further analyses. All other 32 mice showed normal physiological behavior after the procedure and did not require any special treatment during the experiment. The following treatment groups were analyzed: n = 4 apolipoprotein E-deficient (*apoE*^*-/-*^) mice were implanted with bare metal nitinol-stents, n = 5 *apoE*^*-/-*^ mice were implanted with nitinol-stents coated with star-PEG, n = 9 *apoE*^*-/-*^ mice were implanted with star-PEG-coated stents bio-functionalized with RGD and n = 8 *apoE*^*-/-*^ mice were implanted with star-PEG-coated stents bio-functionalized with RGD/CXCL1. All *apoE*^*-/-*^ mice were placed on a high-fat diet containing 21% fat and 0.15% cholesterol (Altromin) after the procedure. C57Bl/6 control mice (n = 6) were implanted with bare metal nitinol-stents and fed a normal chow-diet fed (Ssniff). Mice were sacrificed one week after stent placement under anesthesia (100 mg/kg ketamine hydrochloride and 10 mg/kg xylazine intraperitoneally) by intracardiac puncture and exsanguination, and the carotid arteries were fixed *in situ* with 4% paraformaldehyde. All experiments were conducted in accordance with the guidelines for the care and use of laboratory animals and approved by the Landesamt für Natur, Umwelt- und Verbraucherschutz Nordrhein-Westfalen (permit number 82–02.04.2011.A333) and complied with the German animal protection law. All surgeries were performed under ketamine/xylazin anesthesia and all efforts were made to minimize suffering. Carotid arteries were subsequently embedded in Technovit 9100NEU (Heraeus Kulzer). Serial sections (50μm; 3–5 per mouse) were generated within 500μm from the bifurcation using a cutting-grinding system technique (Exakt 400CS) with a diamond-band saw (Exakt 300) and quantified by Giemsa staining and planimetry of the area within elastic laminae and of the lumen (Diskus software, Hilgers); organized thrombi were detectable by black staining of fibrin depositions inside the neointima. Re-endothelialization of luminal surface was detected after deplasting sections with xylene, 2-methoxyethylacrylate (MEA) and acetone and staining for endothelial cells using vWF mAb (clone A0082, DAKO) and secondary Cy3-conjugated Ab (Sigma Aldrich). Isotype controls (appropriate IgGs) and right carotid arteries served as controls. Images were recorded using a Leica DM-RXE fluorescence microscope and quantified with Diskus software.

### Statistical analysis

Data are expressed as mean±SEM. Statistical calculations were performed using GraphPad Prism 6 (GraphPad Software Inc.). Data were tested for normality (D'Agostino and Pearson omnibus normality test) prior to analysis and non-parametric statistics as applicable. One-way ANOVA analysis followed by the Tukey’s multiple comparison test or, if non-parametric, Kruskal-Wallis test followed by the Dunn’s post-test were applied as appropriate and indicated. *P-values* <0.05 were considered statistically significant.

## Results

**Fig 1:**
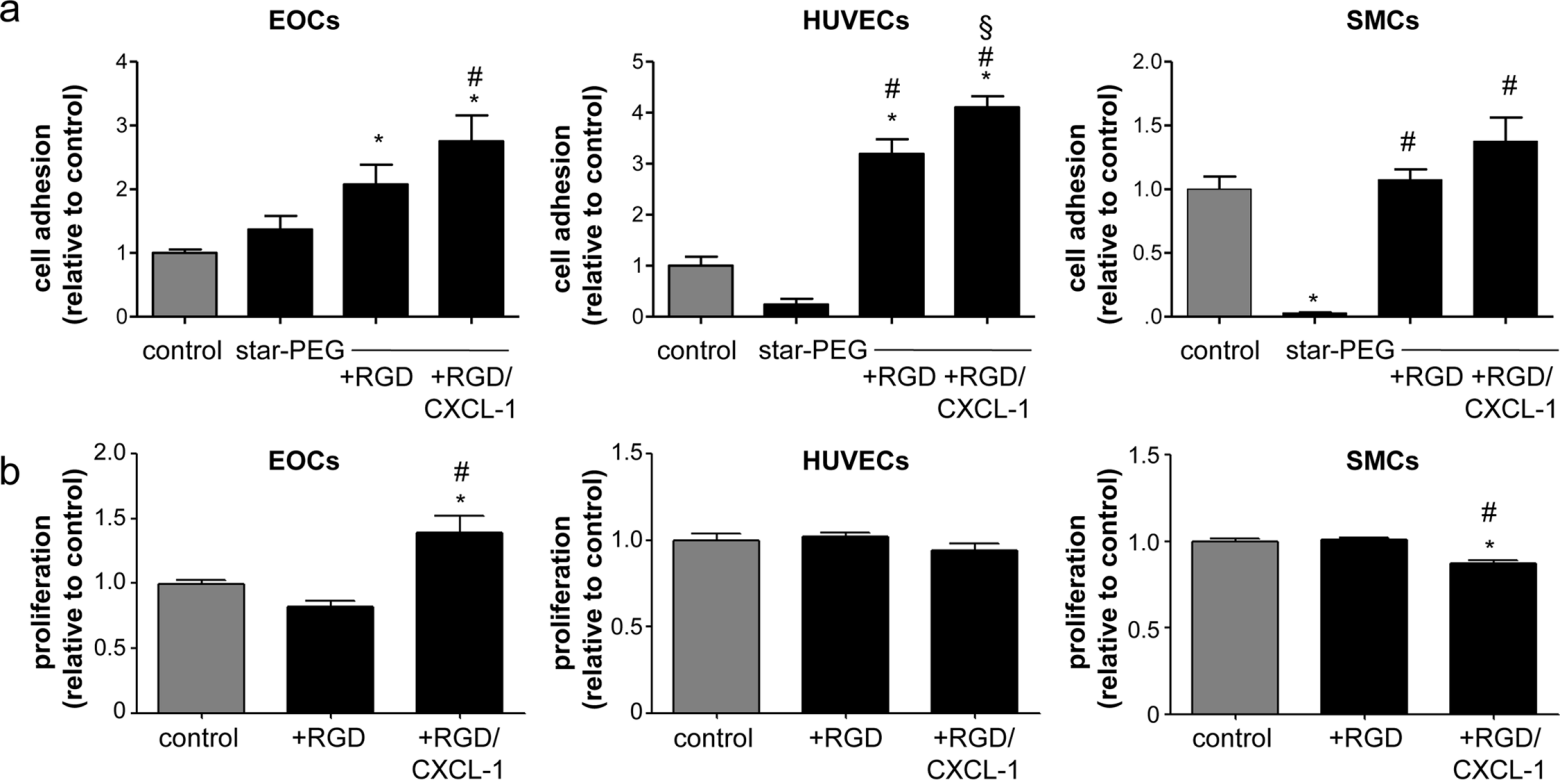
Biofunctionalization with RGD/CXCL1 triggers endothelial cell adhesion. (a) Cells were seeded onto nitinol-foils (control), and nitinol-foils coated with star-PEG, or star-PEG-coated foils bio-functionalized with RGD or RGD/CXCL1. Adherent cells were quantified after 12 hours (n = 10–12). *P<0.05 *vs*. controls, ^#^*P*<0.05 *vs*. star-PEG, and ^§^*P*<0.05 *vs*. star-PEG+RGD-coated foils. One-way-ANOVA with Tukey’s multiple comparison test. (b) Cell proliferation was assessed by BrdU-incorporation (n = 9 each) after indicated stimulation, expressed relative to untreated cells (control). *P<0.05 *vs*. controls and ^#^*P*<0.05 *vs*. RGD. One-way-ANOVA with Tukey’s multiple comparison test.

**Fig 2:**
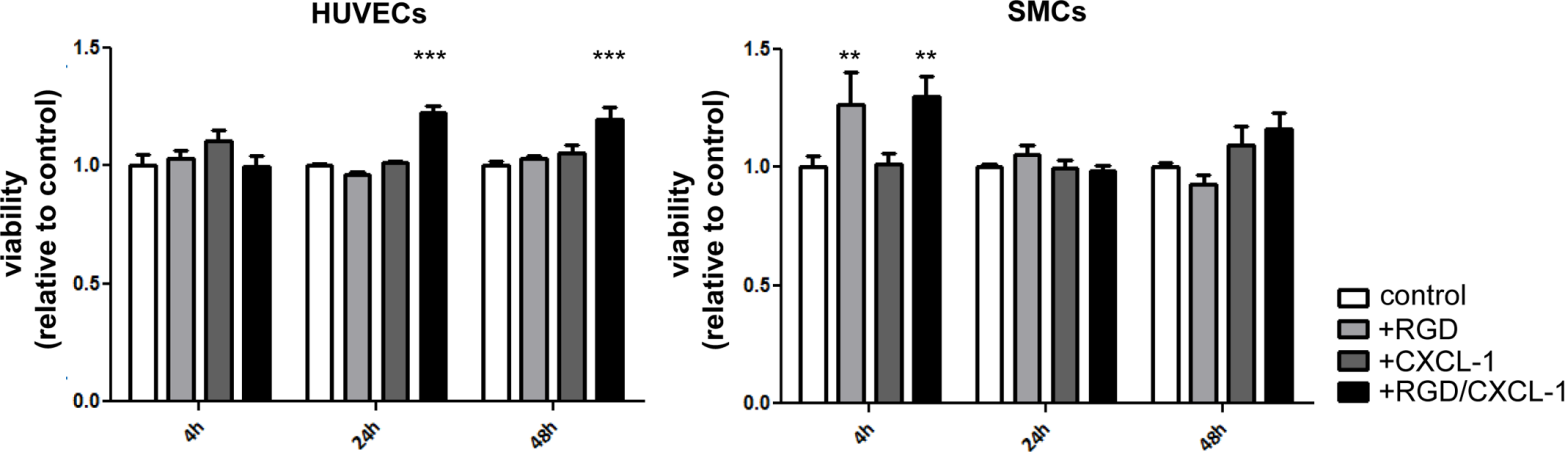
Treatment of HUVECs and SMCs with RGD and CXCL-1 does not impair cell viability. *In vitro* viability assay of HUVECs and SMCs after stimulation with RGD, CXCL-1 or RGD and CXCL-1 together for 4, 24 and 48h. *P<0.05 (shown is significant difference compared to control). Two-way-ANOVA with Bonferroni post-test.

The effects of RGD and CXCL1 on cell adhesion were evaluated when employed as specific surface coatings *in vitro*. EOCs, HUVECs and SMCs were seeded on bare metal nitinol-foils with/without coating with star-PEG, bio-functionalized with RGD, or RGD in combination with CXCL1. A significant increase in EOC and HUVEC adhesion to RGD but foremost RGD/CXCL1-biofunctionalized foils was revealed compared to bare-metal controls or star-PEG-coated foils (Fig 1A). In contrast, SMC adhesion was not altered by RGD/CXCL1 bio-functionalization, and even blocked by star-PEG coating alone (Fig 1A). Stimulation of cells with RGD/CXCL1 significantly augmented the proliferation of EOCs but not HUVECs, whereas proliferation of SMCs was even inhibited compared to untreated cells (Fig 1B). None of these treatments impaired cell viability of HUVECs and SMCs (Fig 2). These data indicate that the combination of RGD/CXCL1 provides support for EOC adhesion and proliferation, and the attachment of HUVECs and SMCs, but not their proliferation.

To scrutinize effects of these coatings *in vivo*, in-stent intima formation was assessed in carotid arteries of *apoE*^*-/-*^ mice one week after stent placement as a model of in-stent stenosis in hyperlipidemic subjects. In contrast to drug-eluting stents for human application, where the kinetics of drug-delivery are of crucial importance, stents used in our study were coated with a polymer, to which RGD and/or CXCL1 were covalently linked, ensuring a densely packed polymer network to produce a RGD and/or CXCL1-coated surface, which keeps its nonfouling properties for at least 7 days [23] and up to 4 weeks at 37°C [24]. We confirmed the stability of the stent coatings after 7-days of washing in PBS under agitation at 37°C by incubation with RITC-conjugated BSA, and could detect an overall unspecific protein binding on only aminofunctionalized stents, whereas no signal could be detected on star-PEG coated stents (data no shown).

**Fig 3:**
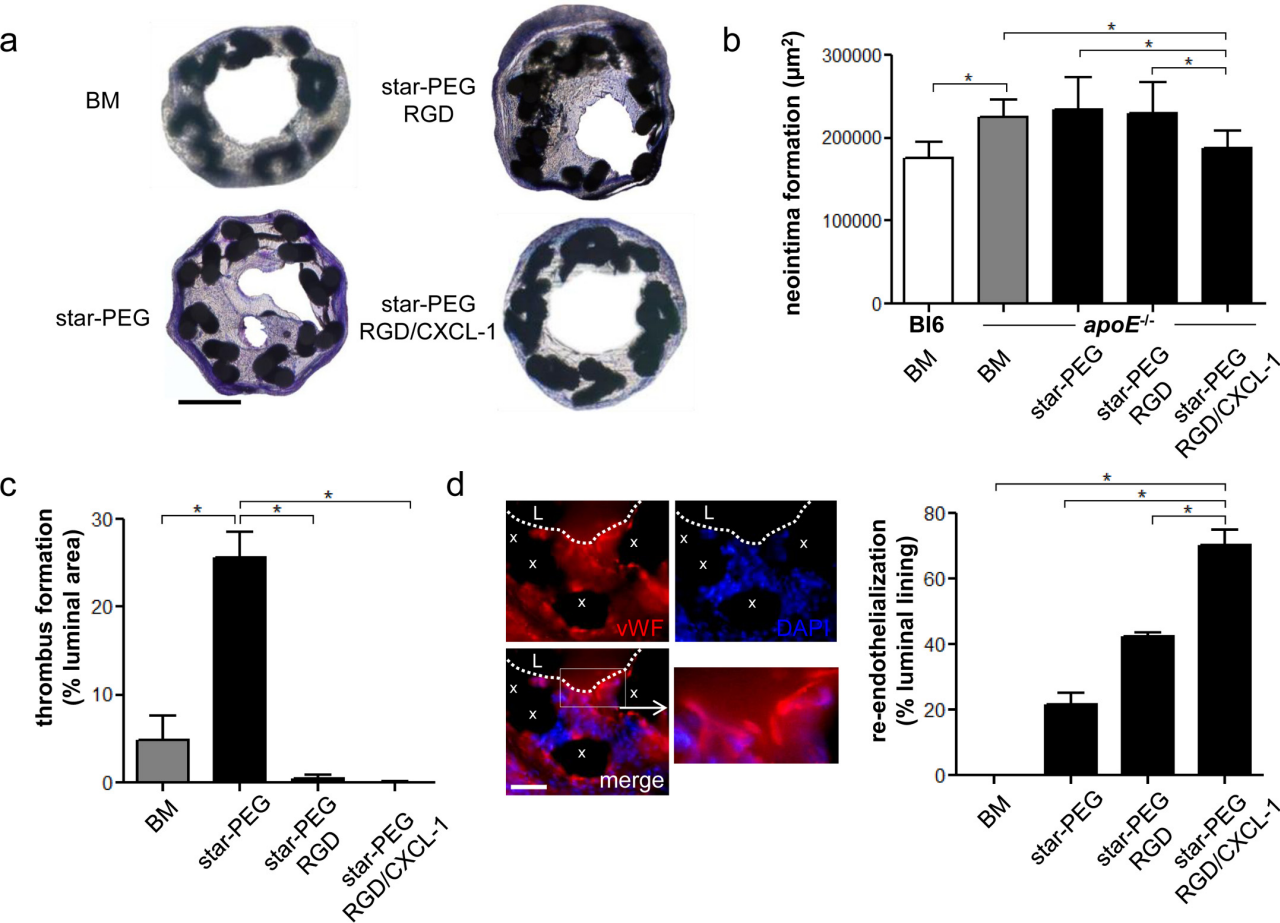
RGD/CXCL1-biofunctionalized stents reduce neointima formation. (a-d) Carotid arteries of C57Bl/6 (n = 6) or *apoE*^-/-^ mice were analyzed one week after implantation of bare metal nitinol-stents (BM, n = 4), star-PEG-coated nitinol-stents (n = 5) or star-PEG-coated stents bio-functionalized with RGD (n = 9) or RGD/CXCL1 (n = 8). (a) Representative Giemsa-stained sections (scale bars, 200μm) from *apoE*^-/-^ mice. Quantification of in-stent intima formation (b) and thrombus formation (c). (d) Representative images of vWF-staining (red); cell nuclei were counterstained by DAPI (L, lumen; x, stent struts; scale bar, 50μm); quantification of re-endothelialization. *P<0.05. Kruskal-Wallis test with Dunn’s post-test.

Compared to C57Bl/6 mice on normal chow diet, the significant increase in in-stent stenosis, evidenced in high-fat diet fed *apoE*^*-/-*^ mice implanted with bare-metal nitinol-stents, star-PEG-coated stents, or stents bio-functionalized with RGD, could be reduced by bio-functionalization with RGD/CXCL1 (Fig 3A and 3B). Thrombus formation was increased in arteries implanted with star-PEG-coated stents, but reduced in RGD-bio-functionalized stents, and almost completely prevented by bio-functionalization with RGD/CXCL1 (Fig 3C). Conversely, re-endothelialization was significantly increased in RGD/CXCL1-bio-functionalized stents (Fig 3D).

These data indicate that bio-functionalizing of star-PEG-coated nitinol-stents with RGD/CXCL1 reduces intima formation after implantation in *apoE*^*-/-*^ mice, associated with an enhanced re-endothelialization.

## Discussion

Revascularizing interventions and stent-implantation lead to a mechanic injury of the endothelial cell lining, inducing thrombocyte aggregation at the injured artery. The administration of thrombocyte aggregation inhibitors is therefore mandatory, but associated with severe bleeding complications [3]. SMC proliferation predominates neointimal hyperplasia at later stages, and is targeted by the drug-eluting stents that inhibit cell proliferation. These therapies, however, can also impair endothelial cell recovery [12], which can precipitate life-threatening late stent-thrombosis. Treatment approaches are therefore required to selectively enhance endothelial recovery after stent-implantation.

We here defined the use of stents coated with a combination of signaling molecules for specifically attracting cells for endothelial repair. We demonstrate that star-PEG bio-functionalization with RGD/CXCL1 effectively supports the adhesion and proliferation of EOCs, and allows the attachment of HUVECs and SMCs, but not their proliferation.

These properties may help to selectively recruit and propagate endothelial cells to cover the stent surface. This notion is supported by *in vivo* evidence showing an accelerated re-endothelialization of RGD/CXCL1-coated stents in carotid arteries of *apoE*^-/-^ mice. In consequence, this may also protect from thrombus formation and enhanced intima formation.

Taken together, our findings indicate that in-stent neointima formation can be reduced by bio-functionalizing star-PEG-coated stents with RGD and CXCL1, harboring the potential of limiting in-stent restenosis in clinical approaches.
